# Successful Handling of Disseminated BCG Disease in a Child with Severe Combined Immunodeficiency

**DOI:** 10.1155/2011/527569

**Published:** 2011-10-24

**Authors:** Sílvia Bacalhau, Cristina Freitas, Rosalina Valente, Deolinda Barata, Conceição Neves, Katrin Schäfer, Annelie Lubatschofski, Ansgar Schulz, João Farela Neves

**Affiliations:** ^1^Pediatric Intensive Care Unit, Hospital Dona Estefânia, 1169-045 Lisboa, Portugal; ^2^Infectology Unit, Hospital Dona Estefânia, Rua Jacinta Marto, 1169-045 Lisboa, Portugal; ^3^Department of Immunology, Rheumatology and Stem Cell Transplantation, University Children's Hospital, 89073-89081 Ulm, Germany

## Abstract

In high-burden countries, *Mycobacterium bovis* Bacillus Calmette-Guérin (BCG) vaccine is administered in newborn to prevent severe *Mycobacterium tuberculosis* infection. Because life-threatening disseminated BCG disease may occur in children with primary immunodeficiency, vaccination strategy against tuberculosis should be redefined in non-high-burden countries. We report the case of a patient with X-linked severe combined immunodeficiency (SCID) who developed disseminated BCG disease, highlighting the specific strategies adopted.

## 1. Introduction

Bacillus Calmette-Guérin (BCG) vaccine is administered worldwide to prevent severe *Mycobacterium tuberculosis* infection. BCG vaccine has an excellent safety profile [1], but a wide range of complications can occur (from simple local lymphadenitis to almost universally lethal disseminated disease) [2]. It is now accepted that most patients with moderate to severe complications have underlying immunodeficiency [3]. Several genetic defects that correlate with BCG disease have now been identified, such as Mendelian susceptibility to Mycobacterial disease (IFN-*γ*-IL-12 pathway), hyper-IgM syndrome, chronic granulomatous disease, and SCID [4]. 

BCG is part of the Portuguese Immunization Program since 1965, being administered at birth to children with weight above 2000 grams. 

We report a patient with SCID with disseminated BCG disease and highlight specific strategies adopted in patient's treatment.

## 2. Case Presentation

A 5-months-old boy was admitted to the intensive care unit of a tertiary pediatric hospital in Lisbon for respiratory insufficiency following three days of fever, cough, and worsening respiratory distress. He presented with oral thrush, bilateral pulmonary rales, and hepatosplenomegaly. He had been vaccinated at birth with BCG (Danish strain 1331) and had had recurrent infections (conjunctivitis, otitis media, and persistent oral thrush). 

There was no consanguinity. Two maternal uncles died of pneumonia in infancy. 

**Figure 1:**
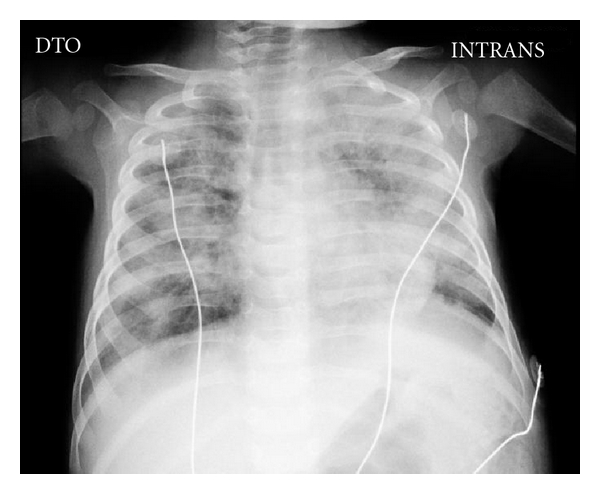
Chest X-ray.

The chest X-ray revealed absence of a thymus and showed bilateral areas of opacities (Figure 1). The immunologic panel confirmed the hypothesis of SCID (Table 1), later genetically characterized (missense mutation in exon 5, codon 226 CGC→CAC, leading to an arginine 226 substitution by histidine; common cytokine receptor gamma chain of IL-2—IL2RG gene). This mutation has previously been described as causing human X-linked SCID [5]. 

Although being asymptomatic, chimerism studies were performed to assess the presence of exogenous maternal lymphocytes, which can be important for an early diagnosis and for the choice of the conditioning regimen if bone marrow transplantation is considered. The results confirmed that 75% of total lymphocyte count was maternal.

The bronchoalveolar lavage was positive for *Candida albicans, Pneumocystis jiroveci,* and acid-fast bacilli. An ultrasound evaluation showed an enlarged spleen with nodules. The BCG primer-specific polymerase chain reaction quickly identified the infection by BCG (amplification of *pncA* gene1 and a multiplex PCR that targets the RD1 region for BCG [6–8]), and presumptive diagnosis of disseminated BCG was made.

He was treated with trimethoprim-sulfamethoxazole and a four-tuberculostatic drug regimen, comprised of isoniazid, rifampin, ethambutol, and levofloxacin, according to laboratory data regarding BCG strain susceptibility. The infant started antibacterial and antifungal prophylaxis and immunoglobulin replacement therapy.

**Figure 2:**
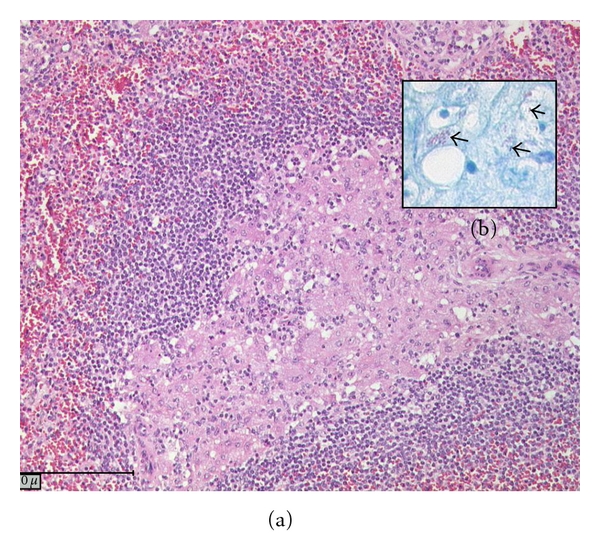
Histologic appearance of spleen sections stained with hematoxylin and eosin (a) and with Ziehl-Neelsen (b) showing BCG infiltration (arrows).

**Figure 3:**
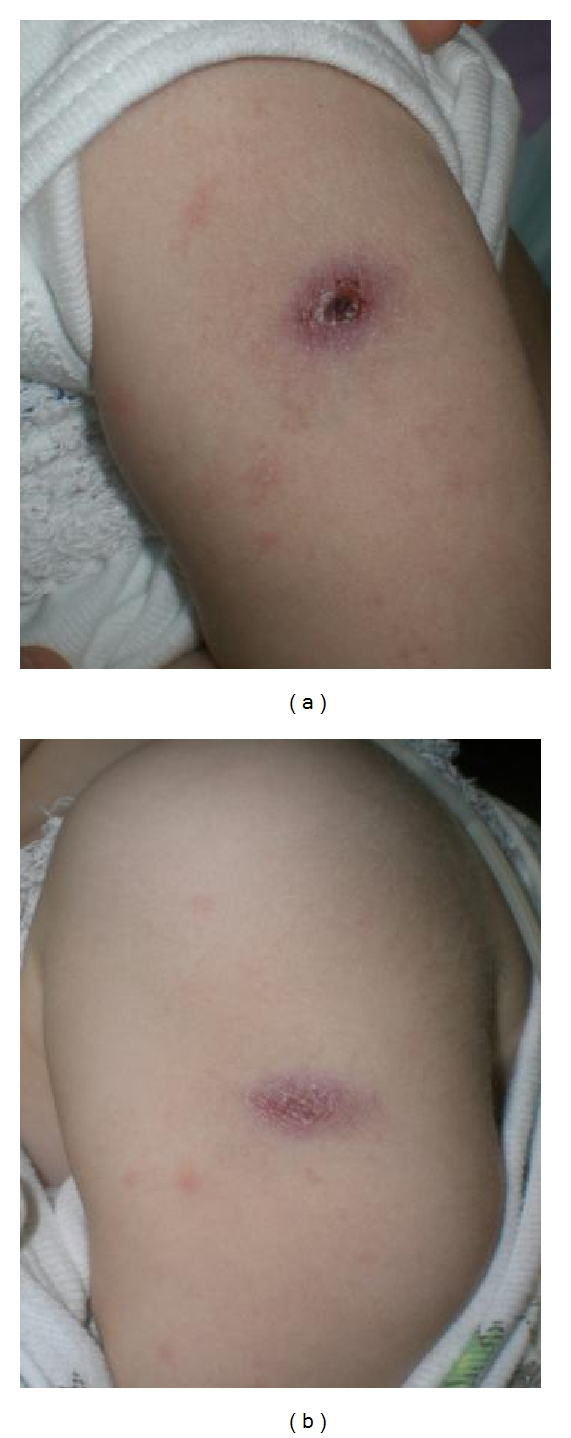
BCG vaccination site at the time of immune reconstitution (a) 5 months after transplantation and (b) 5.5 months after transplantation, showing skin ulceration with massive infiltration of BCG.

Splenectomy was performed aiming for a reduction in BCG load which could worsen the prognosis following immune reconstitution. Spleen analysis revealed severe BCG infiltration (Figure 2), thus establishing the definitive diagnosis of disseminated BCG disease. Four and a half months after the start of tuberculostatic therapy, T-cell depleted bone marrow transplantation (BMT) from an unrelated 10/10 HLA matched donor following reduced intensity conditioning with treosulfan, fludarabine and campath was performed (graft contained 12,5 × 10^6^/kilograms CD34+ stem cells). To enhance immune reconstitution, donor lymphocytes were administered at d + 31, d + 61, and d + 88 containing 5, 15, and 50 × 10^4^ CD3+ T cells per kilograms, respectively. The transplant course was uneventful with the exception of repeated periods of fever that, in the absence of other reported infections, were probably related to active BCG infection, treated continuously by isoniazid, rifampin, and ethambutol. Haematological reconstitution occurred within normal time frame, and there were no signs of graft-versus-host disease. About three months after transplantation, relevant numbers of T cells become detectable. During this phase of immunological reconstitution, signs of BCG infection augmented with prominent nodules at the BCG inoculation site (Figure 3), enlarged thoracic and abdominal lymph nodes, fever, leukocytosis, and massive elevation of C-reactive protein. Linezolid was added to other antituberculous drugs, and some short courses of steroids were administered. The child remained in good clinical condition and is alive and well to date.

## 3. Discussion

We report a case of X-linked SCID with disseminated BCG disease. SCID is the most severe form of primary immunodeficiency disease, characterized by defective T-lymphocyte differentiation that leads to early death in the absence of hematopoietic stem cell transplantation. Patients usually present severe and life-threatening opportunistic infections early in infancy such as disseminated BCG infection [9]. 

Prevalence data of primary immunodeficiency disease (PID) are not available in Portugal. In the USA, the prevalence rate of PID is estimated to be 1 : 2000 children [10] and there are more than 200 cases of disseminated BCG infection in this group of patients [11]. Its estimated incidence is 0.1 to 4.3 per one million vaccinated children and is lethal in 50 to 71% of cases [2, 3, 12]. The availability of the new polymerase chain reaction primers that allow the distinction between *Mycobacterium tuberculosis* and *bovis* is a useful tool in the management of these patients, allowing for quicker diagnosis thus preventing the use of nonappropriate drugs. 

Patients with SCID previously vaccinated with BCG are difficult to manage and should be kept under prophylactic three drug regimens (without pyrazinamide, for which BCG is primarily resistant [13]) after exclusion of disseminated disease and until complete immunologic reconstitution occurs after BMT. 

There are no clear guidelines on the most suitable treatment for disseminated BCG disease [14, 15]. In this case, aggressive therapy (comprising four drugs: isoniazid, rifampicin, ethambutol, and levofloxacin) was administered for four and a half months prior to BMT to reduce the BCG load. For the same reason, splenectomy was performed prior to BMT. Since BCGitis is known to exacerbate clinically during immune reconstitution leading to sepsis and fatal multiple organ failure when T cells rapidly rise after BMT [16], the following modifications of transplant procedure were performed in this case: (a) reduced intensity conditioning using treosulfan and fludarabine instead of the standard regimen (busulfan and cyclophosphamide) to reduce toxicity; (b) T cell depletion of the HLA-identical graft to circumvent BCGitis exacerbation due to rapidly expanding peripheral T cells in the graft; (c) donor lymphocytes in escalating doses to induce a slow but stable T-cell reconstitution; (d) immunosuppression with prednisolone to treat cytokine reactions at the time of overwhelming immune reconstitution. Of course, tuberculostatic drugs have to be administered during the BMT procedure until clinical signs of BCG disappear, which may take several months. In case of empirical treatment, it should be taken into account that BCG vaccine strains differ in their susceptibility pattern to antituberculous drugs and this should be sought. 

In countries where the diagnosis of cellular PID (especially SCID) is well below expected, the possibility of a fatal outcome due to the administration of BCG vaccine in the neonatal period (before the suspicion of cellular PID) is a reality. 

The incidence of tuberculosis in Portugal has been drastically decreasing (from 60 : 100.000 in 1990 to 24 : 100.000 in 2009, of which 5 : 100.000 in children under five years old), and the extrapulmonary forms comprise about 30% of all cases (notably, in 2009 only two cases of “severe” extrapulmonary tuberculosis were reported in children) [17]. This is due to improved public health and social measures, but probably also to the protective effect of BCG vaccination, which is difficult to assess. 

Based on this trend and being aware that the diagnosis of cellular PID remains below expected, the authors underline the need to redefine the vaccination strategy against tuberculosis in Portugal, namely, postponing its administration to the second semester of life in regions where the incidence of the disease is still above 20 : 100.000. 

## Figures and Tables

**Table 1:** Laboratory results. *Maternal T cells in chimerism analysis. ^#^Total chimerism 60% donor, 40% patient. Myeloid chimerism 100% patient, T and NK cell chimerism 100% donor.

Laboratory test	Patient prior to transplantation	Patient 3 months after transplantation	Patient 6 months after transplantation	Reference value
Serum immunoglobulins				
IgA (mg/mL)	<0.1	0.12	0.15	0.2–0.6
IgG (mg/mL)	0,63	Substituted	Substituted	2.3–4.4
IgM (mg/mL)	0,351	0.79	0.32	0.3–0.9

Vaccine titers				
Diphtheria (IU/mL)	0.25	Not done	Not done	>0.5
Tetanus (IU/mL)	0.13	Not done	Not done	>0.5
Isohemagglutinins	Undetected	Not done	Not done	>1/16

Lymphocyte subsets				
CD3+ (cells/*μ*L)	345*	124	1098	2300–6500
CD3+CD4+ (cells/*μ*L)	8	69	960	1500–5000
CD3+CD4+CD45RA (%)	<1	62%	84%	64–92
CD3+CD8+ (cells/*μ*L)	339*	13	91	500–1600
CD3+CD8+CD45RA (%)	0			53–88
CD19+ (cells/*μ*L)	210	1062	3477	600–1300
CD56+ (cells/*μ*L)	18	165	127	100–1000

Lymphocyte proliferation				
Phytohemagglutinin (SI)	3	86		
Anti-CD3 (SI)	1	24		
PPD (SI)	1	9		
V*β* TCR repertoire	restricted	Not done	Not done	
Chimerism	75% maternal	100% donor	60% donor^#^	

Viral loads and PCR				
CMV, EBV, HSV,	All negative	All negative	All negative
HIV1/2,HBV,HCV,			
HHV6/7,VZV,			
Enterovirus, Adenovirus				

PCR Norovirus	Not done	+	+	
